# Dissecting reactive astrocyte responses: lineage tracing and morphology-based clustering

**DOI:** 10.1186/s40659-024-00532-y

**Published:** 2024-08-14

**Authors:** Lina M. Delgado-García, Ana C. Ojalvo-Sanz, Thabatta K. E. Nakamura, Eduardo Martín-López, Marimelia Porcionatto, Laura Lopez-Mascaraque

**Affiliations:** 1 Departamento de Neurobiología Molecular, Celular y del Desarrollo, Instituto Cajal-CSIC, Avenida Dr. Arce 37, 28002 Madrid, Spain; 2 Departamento de Bioquímica, Universidade Federal de Sao Paulo UNIFESP, Rua Pedro de Toledo 669, Sao Paulo, 04039032 Brazil; 3 Present Address: Departments of Neurosurgery and Neuroscience, Yale University School of Medicine, 310 Cedar Street, New Haven, CT 06510 USA

**Keywords:** Astrocyte lineages, Glia biology, Reactive astrocyte response, Morphometrics, Brain injury

## Abstract

Brain damage triggers diverse cellular and molecular events, with astrocytes playing a crucial role in activating local neuroprotective and reparative signaling within damaged neuronal circuits. Here, we investigated reactive astrocytes using a multidimensional approach to categorize their responses into different subtypes based on morphology. This approach utilized the StarTrack lineage tracer, single-cell imaging reconstruction and multivariate data analysis. Our findings identified three profiles of reactive astrocyte responses, categorized by their effects on cell size- and shape- related morphological parameters: “moderate”, “strong,” and “very strong”. We also examined the heterogeneity of astrocyte reactivity, focusing on spatial and clonal distribution. Our research revealed a notable enrichment of protoplasmic and fibrous astrocytes within the “strong” and “very strong” response subtypes. Overall, our study contributes to a better understanding of astrocyte heterogeneity in response to an injury. By characterizing the diverse reactive responses among astrocyte subpopulations, we provide insights that could guide future research aimed at identifying novel therapeutic targets to mitigate brain damage and promote neural repair.

**Graphical Abstract:**

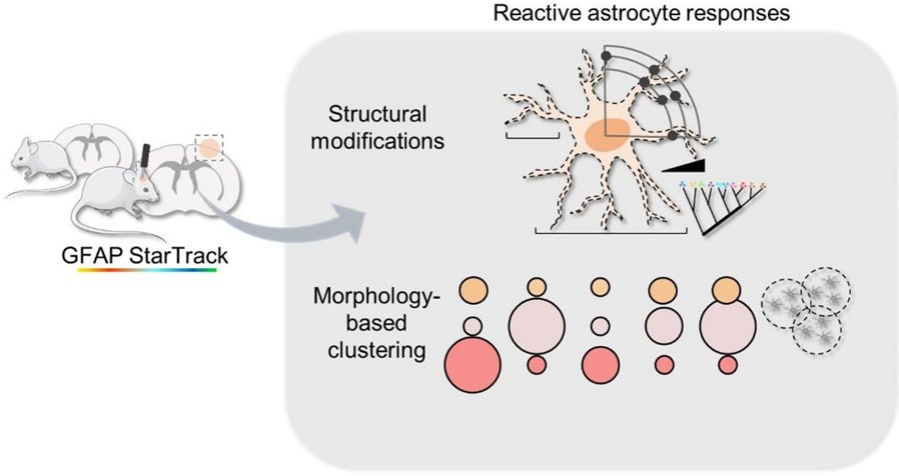

**Supplementary Information:**

The online version contains supplementary material available at 10.1186/s40659-024-00532-y.

## Introduction

Brain damage triggers a complex cascade of cellular and molecular events aimed at restoring homeostasis and neural signaling. Among key cellular players, astrocytes develop neuroprotective or detrimental responses depending on the balance between the gain and loss of their homeostatic functions [18, 57]. Further, the interplay between intrinsic and extrinsic factors, such as the nature and intensity of stimuli, gives rise to diverse reactive responses that might occur simultaneously among astrocyte subpopulations. For instance, the transcriptome of cortical reactive astrocytes in mice subjected to ischemia (middle cerebral artery occlusion, MCAO) differs significantly from those exposed to neuroinflammation (lipopolysaccharide, LPS), which strongly upregulates classical complement cascade proteins associated with neuronal loss [36, 37, 65]. At the molecular level, astrocyte reactivity results in the reorganization of their intermediate filaments, a cytoskeletal network formed by relevant glial proteins such as GFAP, vimentin, and nestin. Increased GFAP content is responsible for characteristic astrocyte responses including resistance to mechanical stress, formation of glial scars, vesicle trafficking, and autophagy [16, 18, 46, 61]. 

However, major questions regarding astrocyte morphological changes in relation to injury remain unknown. These include how astrocytes acquire their shapes, whether and how these changes alter neuron-glia interactions, and whether astrocyte changes contribute to disease causation and progression [4]. Furthermore, the morphological modifications among astrocyte subpopulations, generally known as “astrocyte reactivity”, into the local neuroprotective and reparative signaling within damaged neuronal circuits, continue to be largely unstudied. By elucidating the role of structural modifications in reactive astrocytes, we can advance our knowledge on the biology of glial cells and contribute to the exploration of brain reparative mechanisms at the single-cell level.

The genetic multicolor lineage tracing strategy named StarTrack [24] is a powerful method for genetically and permanently labeling astrocyte progenitors and their progeny, enabling the identification of mature cortical astrocyte clones (reviewed in [9] and [21]). This method is also an effective tool to study morphological and biochemical changes of cortical astrocytes after brain damages such as experimental autoimmune encephalomyelitis (EAE) and traumatic brain injury (TBI) [6, 10, 26, 38]. Previously, we observed that most cortical astrocyte clones exhibited a strong reactive phenotype in response to injury, indicating the presence of intrinsically heterogeneous reactive morphological responses [38]. In this work we took a step further and used the StarTrack method, in conjunction with single-cell imaging reconstruction and multivariate data analysis, to elucidate the underlying reactive responses among different subpopulations of cortical astrocytes. We categorized these astrocyte responses using morphological parameters in a TBI model and provide a comprehensive frame to classify reactive astrocytes based on the intensity of their response to the lesion. Our research involved a comprehensive examination of single-cell modifications in key astrocytic components, coupled with the development of a structure-based classification system for reactive astrocyte responses.

## Results

### Morphological characterization of cortical reactive astrocytes in response to traumatic brain injury

**Fig. 1:**
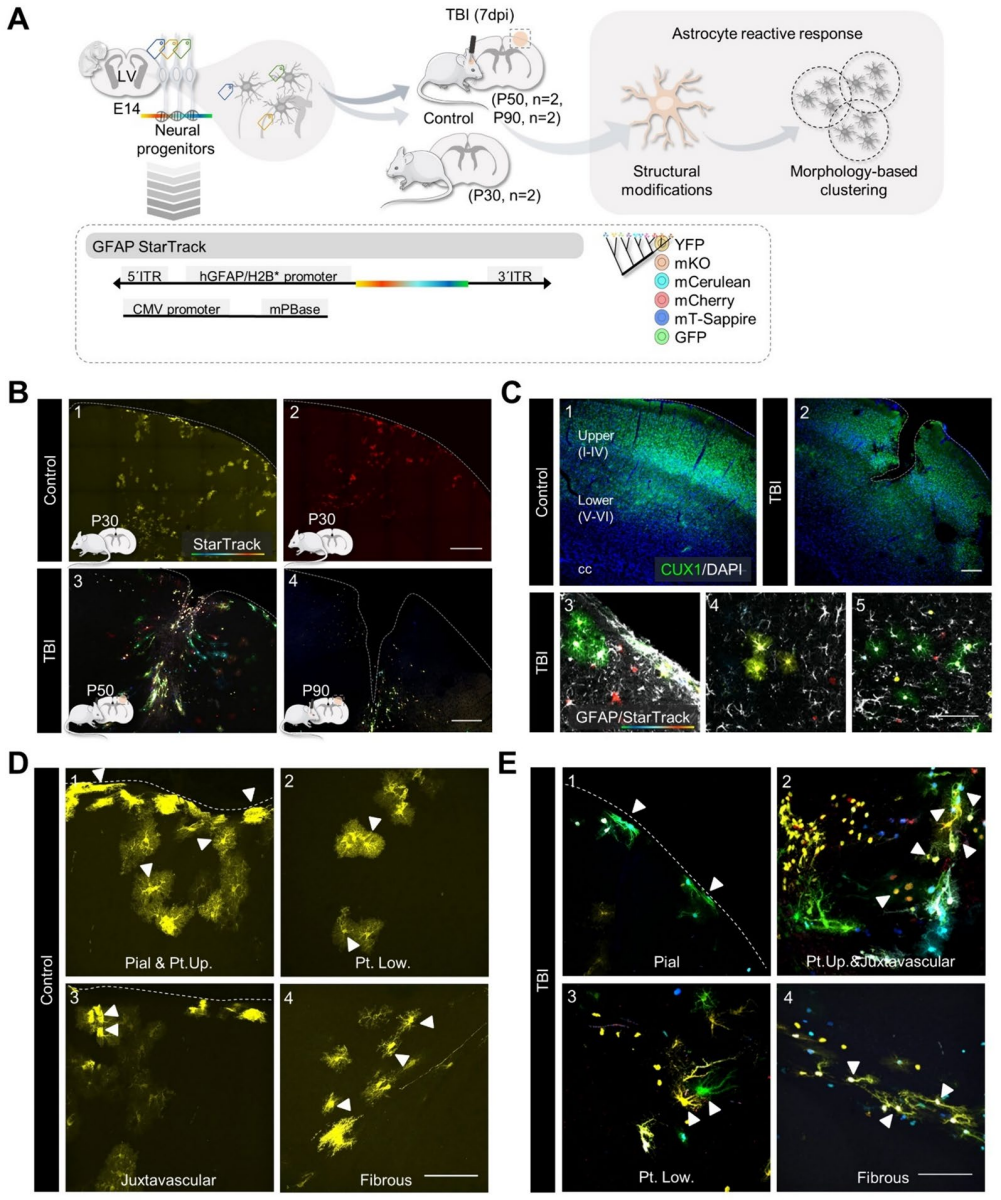
StarTrack reactive astrocyte subpopulations in a model of traumatic brain injury (TBI). **A**. Experimental design. Mice at embryonic day 14 (E14) were in utero electroporated with StarTrack. At postnatal day 50 (P50, n = 2) or 90 (P90, n = 2) mice were submitted to a model of TBI at the somatosensory cortex. Two control mice at postnatal day 30 were included (P30, n = 2). Seven days post-injury (7dpi) we analyzed structural modifications and established a morphology-based clustering method to categorize the reactive responses of astrocytes. **B**. Representative images of the somatosensorial cortex of control (1,2) and TBI (3,4) StarTrack mice. **C**. Representative images of CUX1/DAPI immunohistochemistry. CUX1 was used to delineate upper cortical layers (II-IV; 1,2). Representative images of StarTrack astrocytes, and GFAP immunohistochemistry (3,4,5). The images are from TBI mice. **D and E**. Astrocyte subpopulations along the corpus callosum (cc), cortex (Cx) and *pia mater* (PM) from control (**D**) and TBI (**E**) mice. We identified pial (1), protoplasmic upper and lower layers (1,2,3), juxtavascular (3) and fibrous (4). *TBI* Traumatic brain injury, *Pt. Up.* Protoplasmic upper layers, *Pt. Low.* Protoplasmic lower layers. Scale bar B1-4, 200 μm; C1-5, 200 μm and 4–6, 100 μm; D1-4, E1-4, 100 μm

In this study, we employed the StarTrack genetic lineage tracing method to investigate the heterogeneous reactive responses of cortical astrocytes in a model of traumatic brain injury (TBI). The StarTrack method allows the stochastic expression of six different fluorophores expressed in each of the two main cellular compartments, the cytoplasm, and the nucleus. This gives rise to a total of 12 possible combinatorial expressions of colors, providing a unique ID color code to identify astrocyte clones [24]. Here, we analyzed the structural modifications of control and reactive astrocytes subpopulations and established morphology-based clusters to categorize astrocytes reactive responses (Fig. 1A).

**Fig. 2:**
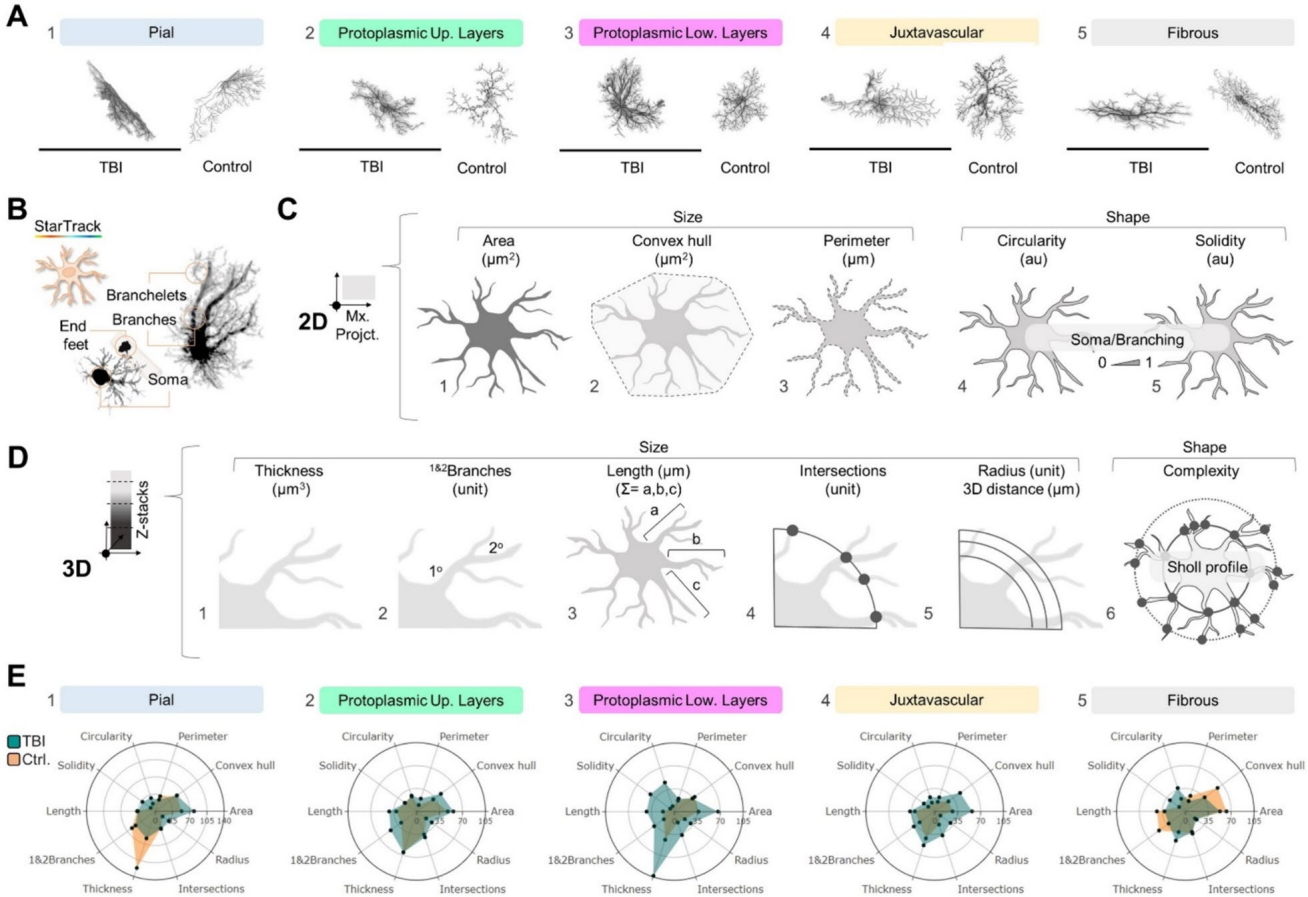
Two- and three-dimensional (2D and 3D) morphometric analysis of astrocyte subpopulations. **A**. Representative images of control and reactive astrocytes subpopulations: pial (1; TBI, n = 15 cells; control, n = 10 cells); protoplasmic upper layers (2; TBI, A1, n = 19 cells, A2, n = 15 cells; control, n = 10 cells); protoplasmic lower layers (3; TBI, A1, n = 13 cells, A2, n = 22 cells; control, n = 10 cells); juxtavascular (4; TBI, n = 19 cells; control, n = 10 cells); and fibrous (5; TBI, n = 15 cells; control, n = 10 cells). Sample size (n) across astrocyte subpopulations. **B**. Representative images of single-cell major astrocytic components -soma, primary and secondary branches, and end feet- across astrocyte subpopulations. **C**. Graphic representation of 2D size- and shape-related parameters: area (1; µm^2^), convex hull area (2; µm^2^), and perimeter (3; µm); 2D-shape related parameters: circularity (4; 4π(“area”)⁄(“perimeter”)^2^) and solidity (5; “area”⁄ “convex hull area”). **D**. Graphic representation of 3D-size and shape-related parameters: thickness (1; relative to 0.05 threshold, µm^3^), 1&2 branches (2; unit), length (3; µm), intersections (4; unit), and radius or 3D distance (5; unit, µm); and 3D-shape related parameter, complexity (6; “intersections” as a function of “3D distance”). **E**. Coefficient of variation (CV) radar graphs across astrocyte subpopulations: pial (1), protoplasmic upper layers (2), protoplasmic lower layers (3), juxtavascular (4), and fibrous (5). Astrocytes subpopulations and parameters were identified as equally significant sources of variability. *TBI* Traumatic brain injury, *Pt. Up.* Protoplasmic upper layers, *Pt. Low.* Protoplasmic lower layers. Scale bar 100 μm

**Fig. 3:**
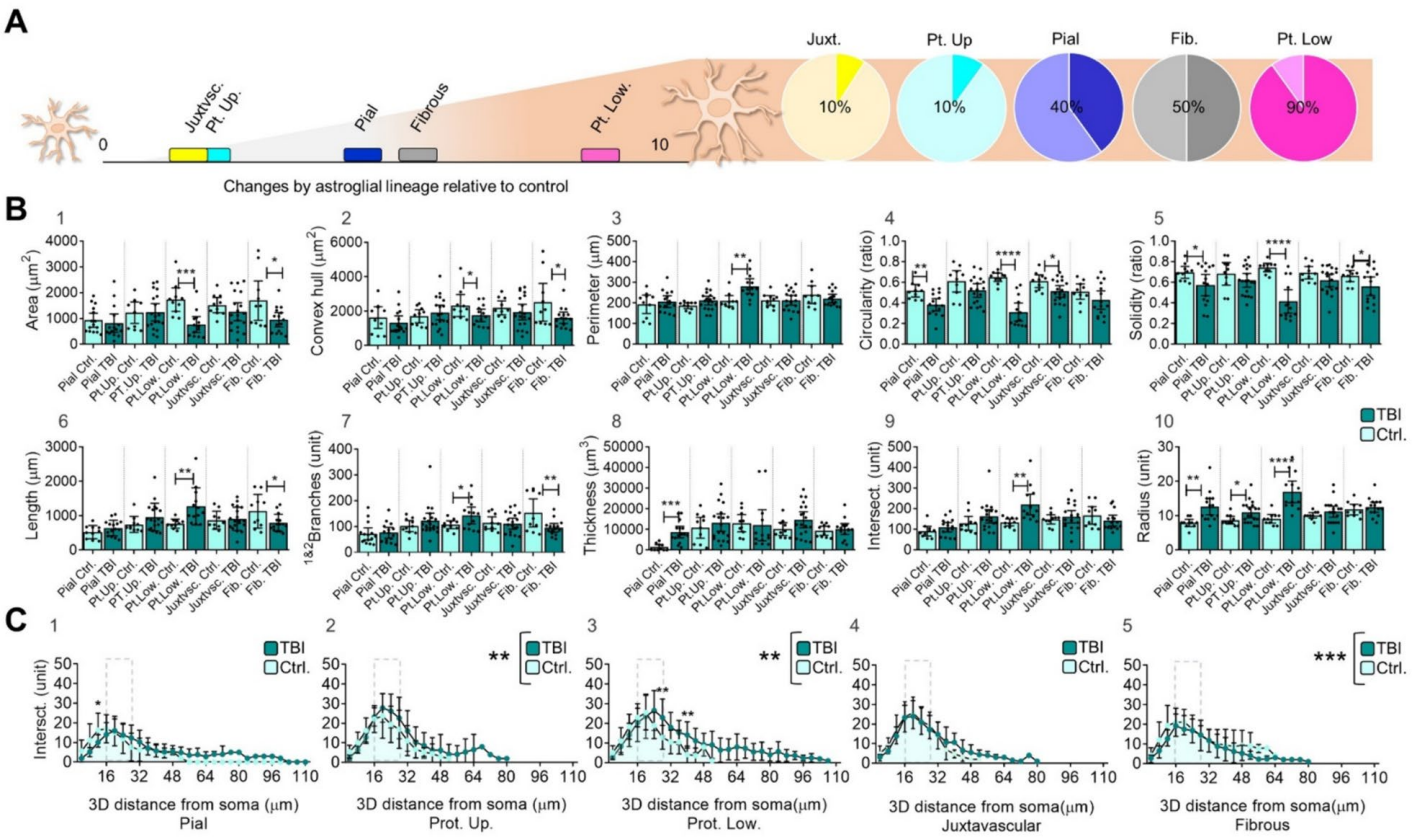
Comparison between control and reactive (TBI) astrocytes. **A**. Proportion of parameters with significant changes across astroglial subpopulations. Lower layer protoplasmic astrocytes exhibited a significant reactive response in nine out of the ten parameters evaluated. The X axis indicates the number of parameters (absolute values) and pie charts their proportion (relative values). **B**. Graph bar of 2D and 3D size- and shape-related parameters in control and TBI reactive astrocytes: area (1), convex hull area (2), perimeter (3), circularity (4), solidity (5), length (6), 1&2branches (7), thickness (8), intersections (9), radius (10). The graph shows the sample´s distribution, mean and 95% CI. Comparison between control and reactive astrocytes was performed by unpaired T test, **** p ≤ 0.0001, *** p ≤ 0.001; ** p ≤ 0.01; * p ≤ 0.05. **C**. XY graph of complexity (Sholl intersections profile) shows the number of intersections along the 3D soma distance. Sholl profile include three segments: an *initial growth segment* extending approximately 12 to 24 μm from the soma, mainly composed by primary branches; followed by a *peak segment* ranging between 16 to 28 μm from the soma (dashed lines) that include the highest number of intersections and their *critical -distance from the soma- value*; and a *final decline segment*, between 24 to 110 μm to the edge of the last branch. The graph shows the mean and standard deviation. Datasets on graphs in absolute values; pial, protoplasmic upper layers, protoplasmic lower layers, juxtavascular and fibrous, control n = 10 cells; pial TBI n = 15 cells; protoplasmic upper layers, TBI n = 19 cells; protoplasmic lower layers, TBI n = 13 cells; juxtavascular TBI n = 19 cells; fibrous TBI n = 15 cells. Comparison between control and reactive astrocytes was performed by two-way ANOVA and Bonferroni´s multiple comparisons test, **** p ≤ 0.0001, *** p ≤ 0.001; ** p ≤ 0.01; * p ≤ 0.05. *TBI* Traumatic brain injury, *Pt. Up.* Protoplasmic upper layers, *Pt. Low.* Protoplasmic lower layers

**Fig. 4:**
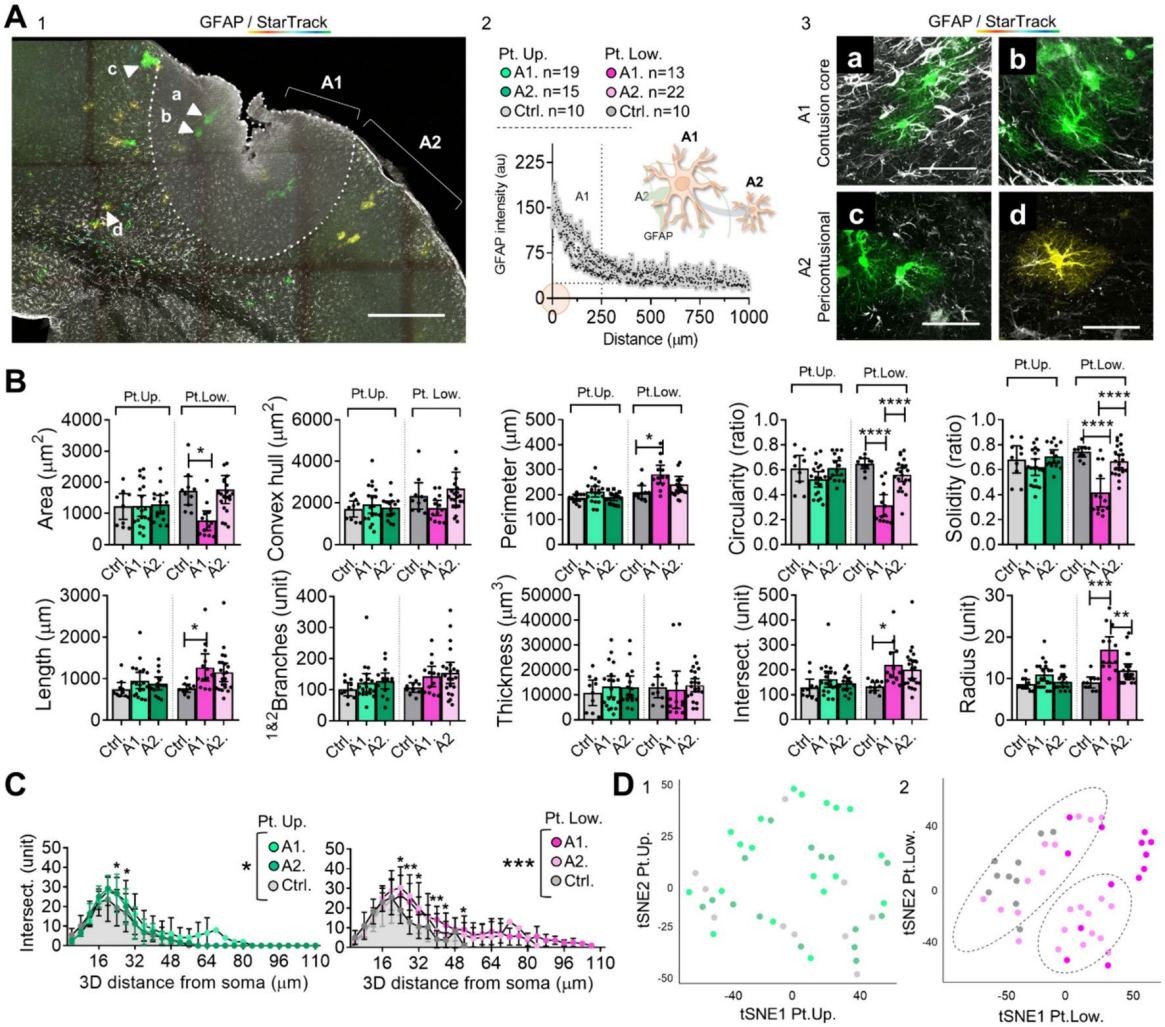
Protoplasmic reactive astrocyte distribution along TBI areas and morphometric analysis. **A**. Representative image of TBI main areas according to GFAP immunolabeling; Pt. Up and Pt.Low (1). Sample size and GFAP fluorescence-intensity profile (2); and representative images of GFAP/StarTrack reactive astrocytes at contusional core (A1; a, b) and pericontusional (A2; c, d) areas (3). The images are from the somatosensory cortex of TBI mice. Fluorescence-intensity profiles revealed an enriched GFAP area (A1) extending approximately 250 μm along the core, followed by a moderate GFAP-normal appearing area (A2). **B**. Graph bar of 2D and 3D size- and shape-related parameters in control, A1 and A2 protoplasmic (upper and lower layers) reactive astrocytes. The graph shows the sample distribution, mean and 95% confidence interval (CI). Comparison between control, A1 and A2 reactive astrocytes (Pt. Up and Pt. Low.) was performed by ordinary one-way ANOVA test and Tukey´s multiple comparisons test, **** p ≤ 0.0001, *** p ≤ 0.001; ** p ≤ 0.01; * p ≤ 0.05. **C**. XY graph of complexity (Sholl intersections profile) shows the number of intersections along the 3D soma distance. The graph shows the mean and standard deviation (SD). Comparison between control and reactive astrocytes (Pt. Up and Pt. Low.) was performed by two-way ANOVA and Bonferroni´s multiple comparisons test, **** p ≤ 0.0001, *** p ≤ 0.001; ** p ≤ 0.01; * p ≤ 0.05. **D**. T-distributed Stochastic Neighbor Embedding (T-SNE) plot and clustering analysis (HDBSCAN) of upper- (1) and lower- (2) layers protoplasmic astrocytes. Datasets on graphs in absolute values; protoplasmic upper layers, control, n = 10 cells, A1, n = 19 cells, A2, n = 15 cells; protoplasmic lower layers, control, n = 10 cells, A1, n = 13 cells, A2, n = 22 cells. Scale A1, 250 μm and A2, 50 μm. *Pt. Up.* Protoplasmic upper layers, *Pt. Low.* Protoplasmic lower layers

**Fig. 5:**
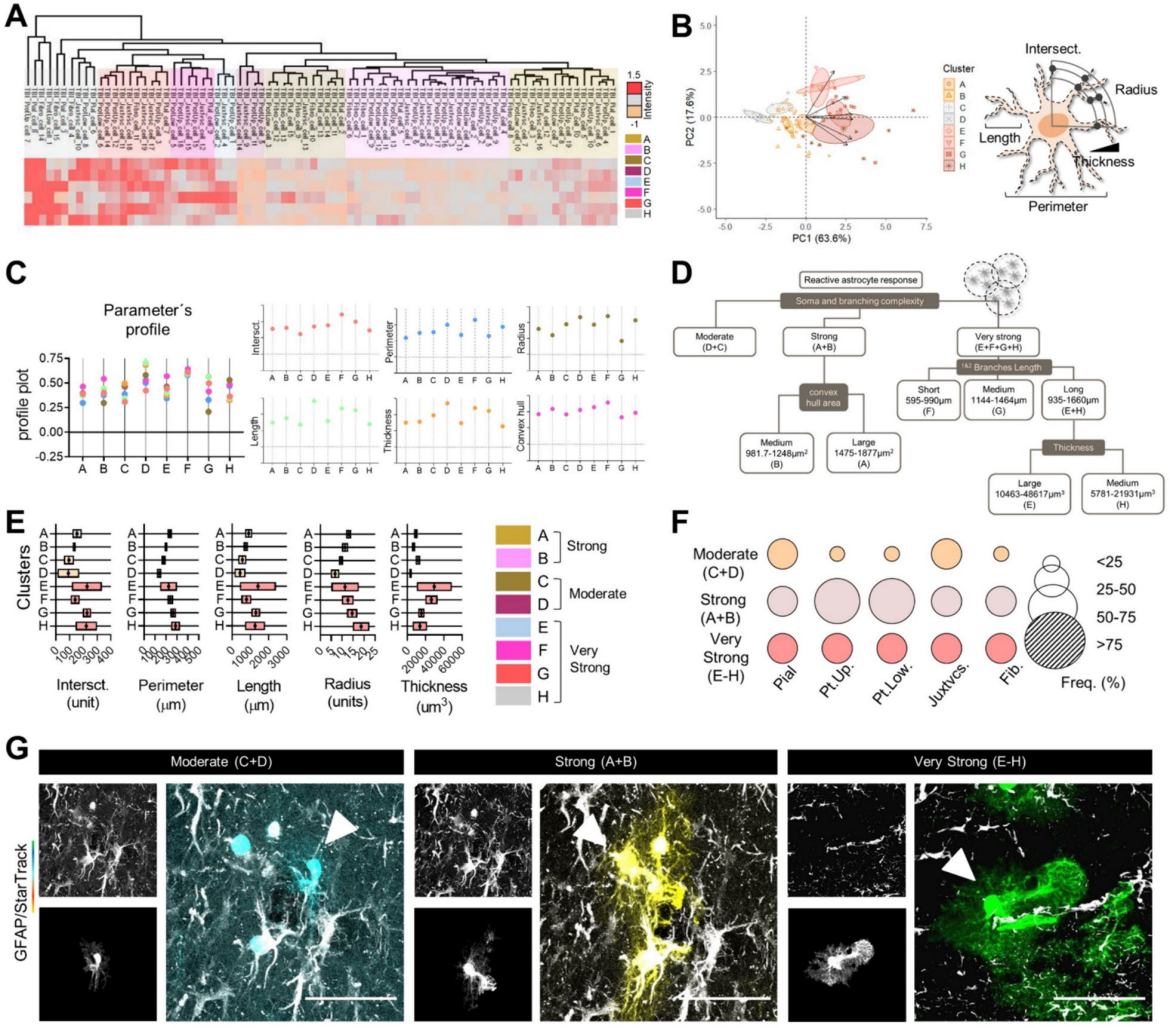
Clustering of reactive astrocytes responses. **A**. Heatmap showing the hierarchical clustering (HC) of reactive astrocytes subpopulations. **B**. Principal component analysis (PCA) plot with confidence ellipses of reactive astrocytes and clusters distribution; graphical representation of selected parameters. **C**. Plot profile (normalized mean) of selected parameters among clusters. **D**. Logical tree of reactive astrocytes response-clusters according to their somatic and branching complexity. **E**. Floating-bars showing the mean and 95% CI for each cluster. **F**. Bubble graph of astrocytes subpopulation frequency among reactive responses. The size of the bubble corresponds to the relative frequency (%). **G**. Representative images of reactive astrocytes responses for juxtavascular subpopulations. Datasets on PCA (**B**), parameters profile (**C**) and cell enrichment graphs (**F**) in relative values, data sets on somatic and branching complexity graphs (**E**) in absolute values. Pial n = 15 cells; protoplasmic upper layers, n = 19 cells; protoplasmic lower layers, n = 13 cells; juxtavascular n = 19 cells; fibrous n = 15 cells. Scale bar 50 μm

**Fig. 6:**
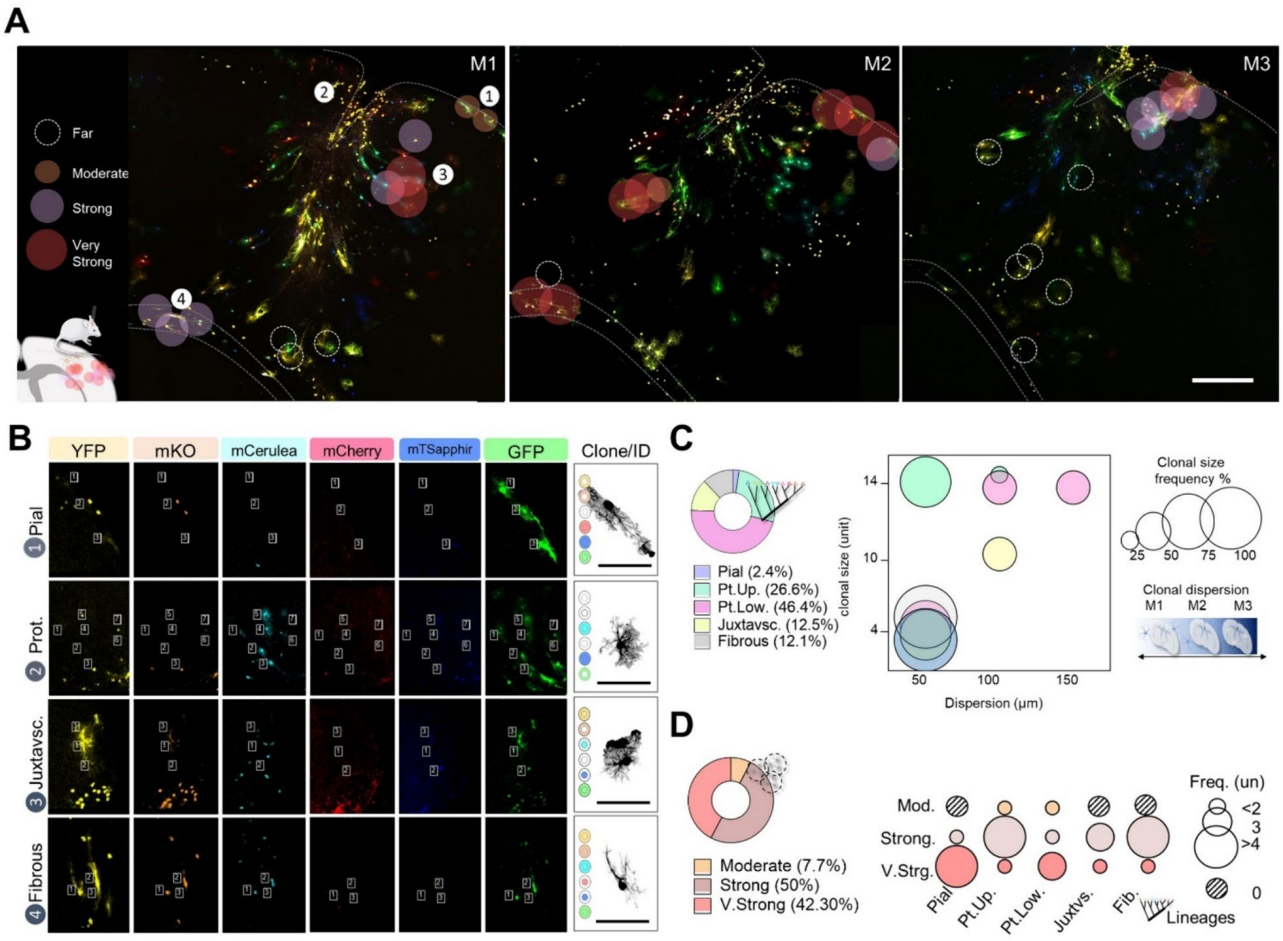
Exploratory analysis of reactive astrocytes responses in animal model of TBI. **A**. Representative TBI sections, astrocytes distribution and reactive responses. **B**. Representative images of the clonal analysis in the TBI model. Astrocytes with the same color code composition were assigned as sibling cells (clones). **C**. Pie chart, and bubble graphs of the frequency and dispersion of sibling cells (clones) among astrocyte subpopulations. Datasets on pie charts in relative values. The size of the bubble corresponds to the relative frequency (%). **D**. Bubble graph of astrocytes subpopulation frequency among reactive responses. Datasets on pie charts in relative values. The size of the bubble corresponds to the absolute frequency (units). Scale bar A, 250 μm; and B, 100 μm

First, we identified distinct astrocyte subpopulations in both control (Fig. 1B, 1–2) and TBI (Fig. 1B, 3–4) groups across the corpus callosum (cc), cortex (Cx) and *pia mater* (PM). To differentiate these subpopulations throughout the Cx, we used CUX1/GFAP immunolabeling to distinguish between upper (layers II–IV) and lower (layers V–VI) cortical astrocytes (Fig. 1C, 1–5). Additionally, we delineated their location within the injury site using GFAP immunolabeling that separate the reactive gliosis area into the “Area 1: contusion core”, and the surrounding normal-appearing tissue termed “Area 2: pericontusional” (Fig. 4A, 1–3). Subsequently, we classified StarTrack labelled astrocytes from both control (Fig. 1D) and TBI (Fig. 1E) groups, based on their localization and morphological properties of their soma and primary branches as follows: (1) pial astrocytes (Fig. 1D, 1 and Fig. E, 1, arrowheads), also known as marginal or perimeningeal astrocytes, located at the surface and in direct contact with the PM; (2) protoplasmic astrocytes (Fig. 1D, 1–2, and Fig. 1E, 2–3, arrowheads), distributed across layers I to VI; (3) juxtavascular astrocytes (Fig. 1D, 3, and Fig. 1E, 2, arrowheads), attached to cortical blood vessels; and (4) fibrous astrocytes in the cc (Fig. 1D, 4, and Fig. 1E, 4, arrowheads). Our analyses included morphological reconstructions of 118 StarTrack reactive astrocytes (n = 4, Fig. 2A, 1–5 TBI), and 45 StarTrack astrocytes that were used as controls (n = 2, Fig. 2A, 1–5 Control). For each cell, we identified major astrocytic components, soma, and primary and secondary branches (branchlets), to analyze their morphological profile (Fig. 2B). Thus, we employed two- and three-dimensional (2D and 3D) image projections (Fig. 2C and 2D) to assess size- and shape-related parameters such as “cell body area” (μm^2^; Fig. 2C, 1), “convex hull area” (μm^2^; Fig. 2C, 2), “perimeter” (μm; Fig. 2C, 3), “circularity” (4π(“area”)/(“perimeter”)^2^; Fig. 2C, 4), “solidity” (“area”/”convex hull area”; Fig. 2C, 5), total “thickness” (μm^3^; Fig. 2D, 1), “1&2 branches” (unit; Fig. 2D, 2), total branches “length” (μm; Fig. 2D, 3), “intersections” (unit; Fig. 2D, 4), “radius” or “3D distance” (unit, μm; Fig. 2D, 5) and “complexity” (Fig. 2D, 6). Altogether, this dataset let us to explore astrocyte territories and branching: parameters such as “area”, “perimeter” and “convex hull area”, measured the size of the soma and branches of astrocytes (Fig. 2C, 1–3), while parameters such as “circularity” and “solidity” (Fig. 2C, 4–5) measured their general shape variation. These shape-related parameters utilized ratios to compare the “area” of each astrocyte with its “perimeter”, in the case of “circularity”; and with its “convex hull area”, in the case of “solidity”. Higher values of “perimeter” and “convex hull area” resulted in lower “circularity” and “solidity” ratios, that indicated “less regular” and “less dense or spongiform” astrocyte morphologies. The parameter “thickness” (Fig. 2D, 1) measured the total astrocyte volume, although this parameter is mainly affected by astrocyte branching, as their processes constitute up to 95% of total cell volume [64]. Similarly  parameters “1&2 branches” and “length” (Fig. 2D, 2–3) determined cell branching. In addition, we included Sholl analysis metrics such as “intersections”, “radius” or “3D distances”, and “complexity” or “Sholl intersections profile” (Fig. 2D, 4–6).

By analyzing these datasets of parameters, we constructed radar graphs with coefficient of variation values (CVs) for each parameter to gain insights into the biological trends among control and TBI groups (Fig. 2E). Interestingly, we found that both, astrocyte subpopulations and morphological parameters contributed equally to variability. Astrocyte variability ranged from 7.70% (CV < 10%, indicating low variability) in control protoplasmic “solidity” (lower layers, Fig. 2E, 3) to 121.8% (CV > 35%, indicating high variability) in pial “thickness” (Fig. 2E, 1). Specifically, subpopulations exhibiting acceptable interindividual variability included control protoplasmic (upper and lower layers; CV < 35%, acceptable; Fig. 2E, 2–3), and juxtavascular astrocytes (Fig. 2E, 4), as well as TBI fibrous reactive astrocytes (CV < 35%, acceptable; Fig. 2E, 5). Parameters with acceptable CV included “perimeter”, “circularity”, “solidity”, “length”, “intersections”, and “radius”, which were efficient in detecting significant changes between control and TBI groups (Fig. 2E).

Comparisons between control and TBI groups revealed that lower layer protoplasmic astrocytes exhibited significant changes in nearly all size- and shape-related parameters, including “area”, “convex hull”, “perimeter”, “circularity”, “solidity”, “length”, “branches”, “intersections”, and “radius” (Fig. 3A, B, 1–10). In contrast, upper layer protoplasmic astrocytes only exhibited significant changes in the size-related parameter “radius” (also interpreted as the “3D distance” (Fig. 3A, and Fig. 3B, 1–10). Distinct responses were also observed among juxtavascular, pial and fibrous astrocytes. Juxtavascular astrocytes showed only significant changes in shape, as measured by “circularity” (Fig. 3A and Fig. 3B, 1–10). Pial and fibrous reactive astrocytes, however, showed changes in both, shape- and size-related parameters: pial astrocytes showed alterations in “circularity”, “solidity”, “thickness” and “radius”, while fibrous astrocytes showed alterations in “area”, “convex hull area”, “solidity”, “length”, and “branches” (Fig. 3A and, Fig. 3B, 1–10). Upon a more comprehensive analysis of the datasets from both the control and TBI groups, we found that under physiological conditions (control group), only the pial lineage exhibited a distinct profile among astrocyte subpopulations. However, following TBI, lower layers protoplasmic astrocytes displayed differential profiles in shape- and size-related parameters across astrocytes subpopulations (data not shown).

Further analysis of “complexity” (Sholl intersections profile) allowed us to identify three distinct segments in astrocyte branching (Fig. 3C, 1–5). These segments included: (1) an *initial growth segment* extending approximately 12–24 μm from the soma, mainly composed by primary branches; (2) a *peak segment* ranging between 16 and 28 μm from the soma (dashed lines) that included the highest number of intersections and their *critical -distance from the soma- value*; and (3) a *final decline segment*, with a size ranging between 24 and 110 μm to the edge of the last branch. Comparisons between control and TBI groups revealed changes in astrocyte branching density and spatial distribution for protoplasmic (upper: Fig. 3C, 2 and lower: Fig. 3C, 3) and fibrous (Fig. 3C, 5) astrocytes. In particular, protoplasmic reactive astrocytes increased their complexity while fibrous reactive astrocytes showed an opposite response (Fig. 3C). Collectively, our findings highlight distinct profiles among astrocytes subpopulations, revealing specific size- and shape- related alterations in response to the brain damage. The morphological changes observed in upper- and lower-layers protoplasmic astrocytes prompted us to further investigate the relationship between astrocyte reactivity and their spatial distribution.

### Influence of the distance to the lesion in reactive astrocytes morphologies

Protoplasmic astrocytes were analyzed based on their location within the cortical layers (see above, Fig. 1C, 1–2) and their distance from the injury, as determined by GFAP immunolabeling. This analysis separated the area of reactive gliosis, named as “Area 1: contusion core”, from the surrounding normal-appearing tissue, named as “Area 2: pericontusional” (Fig. 1C, 3–5 and Fig. 4A). Areas 1 and 2 were stablished using image analysis extending 1000 μm from the injury site, revealing GFAP fluorescence-intensity profiles. This led to define two main areas: Area 1 (A1), characterized by an increased GFAP intensity profile extending approximately 250 μm from the injury site, and Area 2 (A2), immediately adjacent to A1, with a lower GFAP intensity profile (Fig. 4A, 1–2). A1 was identified as a reactive gliosis region, while A2 had a GFAP-normal-appearing profile (Fig. 1C, 3–5 and Fig. 4A, 3).

Our analysis revealed that only A1 lower-layers protoplasmic astrocytes showed distinct reactive responses compared to A2 astrocytes (Fig. 4B). These responses included morphological alterations in both size  and shape-related parameters, such as “area”, “perimeter”, “circularity”, “solidity”, “length”, “intersections”, and “radius” (Fig. 4B). Interestingly, the analysis of “complexity” (Fig. 4C) indicated a group effect for both—control and reactive astrocytes in upper and lower cortical layers, with a particular emphasis on lower-layers astrocytes that displayed larger 3D distances, and complexity-curve shapes with a more regular decline as they moved away from the soma (Fig. 4C).

Pairwise analysis of each shape- and size-related parameters, along with visualization of the cell population in t-distributed Stochastic Neighbor Embedding (t-SNE. Figure 4D) plots, revealed that A1 and A2 upper layers protoplasmic control and reactive astrocytes were randomly distributed in the plot (Fig. 4D, 1). In contrast, most A1 lower layers protoplasmic astrocytes were distinctly clustered. On the other hand, A2 lower layers protoplasmic reactive astrocytes were distributed next to controls, indicating similar morphological profiles (Fig. 4D, 2). Consistent with these observations, further clustering analysis based on the TSNE distribution (Fig. 4D, 2) grouped A2 lower-layers protoplasmic reactive astrocytes with controls.

### Reactive response landscape among astrocyte lineages

Lastly, we integrated size- and shape-related parameters to construct morphology-based clusters of reactive responses and explored astrocyte lineage participation (Fig. 5). Initially, we applied a multimodal index analysis (MMI, Suppl. Figure 2A) to guide the selection of parameters for cell clustering, focused on those with MMI´s values greater than 0.55, such as “convex hull area”, “perimeter”, “length”, “thickness”, “intersections”, and “radius” (Suppl. Figure 2A). Subsequent hierarchical clustering (HC, Fig. 5A and Suppl. Figure 1), and principal component analysis (PCA, Fig. 5B) enabled the partition of the dataset into clusters labeled from A to H, which were then categorized based on their reactive responses (Fig. 5A, B and Suppl. Figure 2B). Analysis of variance (ANOVA) confirmed that clusters A-B and C-D exhibited similar parameter profiles, while clusters E, F, G, H displayed greater variability (Fig. 5C and Suppl. Figure 2B). Principal component 1 (PC1, 63.6%) and Principal component 2 (PC2, 17.6%) collectively explained 81.2% astrocyte variation in a bidimensional distribution, with optimal correlation values among parameters (Fig. 5B and Suppl. Figure 2C, 1). PC1 included measurements of the size-related parameters “length”, “intersections”, and “perimeter”; PC2 predominantly received contributions from “thickness” and “radius”. The PC biplot displayed how selected morphological parameters influenced and contributed to the direction of the datasets (Suppl. Figure 2C, 1). Upon conducting a PC biplot on relevant datasets, it became evident that the distribution of the plot was influenced by upper- and lower-layers protoplasmic astrocytes (Suppl. Figure 2C, 2).

Next, we built a dendrogram (logical tree) to establish diversification nodes for astrocyte reactive responses based on astrocyte clusters, individual features, and type of reactive responses (Fig. 5A–D). The dendrogram was constructed using a hierarchical clustering approach that identified diversification nodes based on a 95% confidence interval (CI) for each parameter to suggest cut-off values (Fig. 5D, E). Parameters such as “perimeter”, “intersections”, and “radius” defined the first-level nodes of the tree. Parameters like “length” and “convex hull area” were used to determine second- and third-level branches nodes, while “thickness” defined fourth-level branches and terminal nodes (Fig. 5D). For the first-level nodes, reactive astrocytes responses were classified into 3 group nodes: “moderate” node, which included astrocytes from clusters C and D (C + D); “strong” node, included clusters A and B (A + B); and “very strong” node that clustered the E to H astrocyte groups (E, F, G, H) (Fig. 5D). Clusters A and B could be further subdivided into in “medium” and “large” second level nodes based on their “convex hull area” values, while clusters E to H, (E, F, G, H) could be subdivided into “short”, “medium”, and “long” based on their processes “length”, and into “large” and “medium” based on their “thickness” values. Interestingly, the reactive response type “very strong” consisted of clusters with greater variability (E, H), primarily characterized by extreme “length” and “thickness” values (Fig. 5D).

We also examined astrocyte diversity within each reactive response (Fig. 5F). The “strong” reactive response (A + B) had the highest proportion of protoplasmic astrocytes from both upper and lower layers, while the “very strong” type exhibited similar lineage participation (Fig. 5F and Suppl. Figure 3D). This diversity was evident through the StarTrack method and astrocytes reconstructions, which allowed us to group different responses based on similar morphologies (Fig. 5G and Suppl. Figure 2D, 1–2). For example, reconstructions and categorizations of reactive astrocytes in two TBI mice (A147 and A117 mice; aged 50, and 90 days respectively) revealed 2 main response models: Model 1 (enriched with “strong” and “very strong” reactive response, primarily from pial, upper-layers protoplasmic and fibrous lineages (Suppl. Figure 2D, 1), the Model 2 displayed proportional types of reactive responses, enriched with pial (moderate), protoplasmic low (strong) and juxtavascular (very strong) lineages (Suppl. Figure 2D, 2).

Finally, we conducted an exploratory analysis to characterize the morphology of different astrocyte responses grouped in our dendrogram using one TBI mouse (A147). This analysis integrated clonal analysis, categorization of reactive responses in astrocyte subpopulations, and their spatial distribution. Our findings revealed that similar reactive responses were localized proximally and at similar distances from the injury (Fig. 6A). Clonal analysis of 281 astrocytes, corresponding to 35 clones with identical fluorophore combinations in both the nucleus and cytoplasm (Fig. 6B), showed that all types of astrocyte subpopulations -pial, upper- and lower- layers protoplasmic, juxtavascular, and fibrous- were present around the injury site. Among these, upper- and lower- layers protoplasmic astrocytes were the most prevalent, followed by fibrous astrocytes, while juxtavascular and pial astrocytes represented smaller fractions (Fig. 6C). Quantitative analysis of the number of sibling cells showed variability in clone size and dispersion across astrocyte subpopulations (Fig. 6C). Additionally, the frequency analysis of astrocyte subpopulations indicated that the “strong” type of reactive response (A + B) had the highest proportion of protoplasmic (upper layers) and fibrous astrocytes. In contrast, the “very strong” reactive response exhibited the highest proportion of pial astrocytes (Fig. 6D). Overall, our data provide a framework for detecting morphological similarities and differences among astrocyte subpopulations, suggesting that TBI induces distinct morphological signatures in reactive astrocytes.

## Discussion

In this work we explored specific subpopulations of StarTrack-labeled astrocytes following traumatic brain injury, focusing on their morphology-dependent responses. Our study revealed intricate single-cell modifications within critical astrocytic components -soma, branches and branchlets- highlighting significant variability in reactive responses among astrocytes subpopulations.

Astrocyte research encompasses various dimensions including regional, molecular, and biochemical aspects, developmental stages, interspecies adaptations, aging, brain damage, and neurological conditions [7, 17–19, 32, 39, 45]. From pioneering works in glia biology [47, 53, 59] to recent state-of-the-art reviews [4, 17, 57] it is now well-established that the size, shape, and complexity of astrocytes are closely linked to their functional status and their ability to interact with diverse neural and non-neural cells. Here, we identified distinct subpopulations of StarTrack-labeled astrocytes by examining their functional roles, regional distributions, and cell-lineages. Based on their localization within cortical regions and layer-specific interactions, we classified these StarTrack-labeled astrocytes into pial, protoplasmic (both upper- and lower-layers), juxtavascular, and fibrous subpopulations. Research has highlighted the significant role of astrocyte morphology in their specialized functions. For instance, human protoplasmic astrocytes are characterized by highly complex branching morphologies, which are closely linked to the regulation of synaptic communication. In contrast, fibrous astrocytes display simpler branching patterns and primarily contributed to structural support [15, 29, 35, 41, 56]. Additionally, interactions with the vascular network are crucial for the functions of perivascular and juxtavascular astrocytes. Perivascular astrocytes, which extend their end-feet to blood vessels, and juxtavascular astrocytes whose cell bodies are closely associated with blood vessels, play roles in vascular regulation and maintenance [5, 25, 33, 43, 58].

Once we established the morphological characteristics of reactive astrocytes, our next step was to explore the distinct morphological alterations among astrocytes subpopulations in response to injury. Currently, methods for assessing astrocyte morphology often face limitations due to the constraints of microscopy, particularly in resolving the full complexity of 3D branching structures, including finer processes like branchlets and leaflets [4]. In this study, despite facing interindividual variability values in most size-related parameters (indicated by coefficient of variation, CV values), our approach -combining StarTrack labeling with detailed morphological analysis-enabled a pioneer method  to correlate changes in astrocyte morphology with response to injury. By analyzing shape, size, and their interplay, we provided a comprehensive framework to understand how these morphological features impact the intensity of astrocyte responses to injury. This approach highlights the importance of considering astrocyte morphology as a complex and multifaceted set of features, rather than isolating individual parameters.

Indeed, while shape-related parameters such as “solidity,” “circularity,“ and “complexity” are less frequently analyzed compared to size-related parameters, they proved to be highly effective in detecting differential reactive responses among astrocyte subpopulations. These shape-related parameters served as global descriptors, measuring the extent of changes in astrocyte shapes. For instance, in our study, upper and lower protoplasmic reactive astrocytes exhibited reduced circularity and solidity ratios, indicating more irregular and spongiform cell bodies, along with enriched Sholl profiles (complexity), which described cell branching density and spatial distribution. These variations likely reflect the adaptation of protoplasmic astrocytes to the characteristic loss of cell polarity and disruption of tissue organization within the damaged neural network at the primary injury site. Following a TBI, many processes such as cell death, neuroinflammation, cell proliferation, and tissue repair overlap to one another, resulting in loss of astrocyte subpopulations and the formation of astrocyte borders, where newly formed astrocytes demarcate and separate damaged areas from the healthy surrounding tissue [11, 22, 60]. Interestingly, we found that not only protoplasmic astrocytes, but also pial and juxtavascular astrocytes experienced morphological changes in response to the injury (reactivity) that integrated shape- and size-related parameters. These reactive astrocytes exhibited “more irregular” and “spongiform” cell bodies (lower circularity and solidity values) and increased thickness. The increase in thickness, a size-related parameter, has been associated with the enrichment of cytoskeletal content or cell swelling, typical from the hypertrophy of reactive astrocytes [34, 49]. The increase in the cell body volume may be linked to intense modulation of blood–brain and blood-cerebrospinal fluid barriers function [34]. TBI causes massive rupture of the meninges, blood vessels, and neural tissue, compromising blood–brain barriers and leading to astrocyte reactivity and modulation of vascular permeability and the movement and exchange of molecules [30, 48].

Subsequently, we explored the relationships between astrocyte reactivity and their spatial distribution in upper- and lower-layer protoplasmic astrocytes. Lower-layer protoplasmic astrocytes exhibited significant reactive response in nine out of the ten parameters evaluated, while upper-layer protoplasmic astrocytes showed significant changes in only one parameter. To delineate areas of reactive gliosis (A1 or contusion area) from GFAP-normal-appearing astrocyte profiles (A2 or pericontusion area), we employed a GFAP antibody to identify the extension of the lesion in the brain parenchyma. This approach is consistent with previous studies in both human and mouse models, which have divided the damaged region to investigate specific cellular and tissue aspects. These investigations addressed molecular aspects [27, 40], vascular and perfusion dynamics [55], metabolic changes [63], and clinical progression [1].

Our analysis revealed distinct morphological variations in protoplasmic astrocytes based on their cortical layer and proximity to the injury site. Specifically, upper-layer protoplasmic astrocytes exhibited relatively stable morphologies across different injury areas (A1 or A2), lower-layer protoplasmic astrocytes demonstrated pronounced and differential morphological profiles between these areas. This disparity was further emphasized by TSNE and clustering analyses, which showed that reactive lower-layer astrocytes from A2 had morphological profiles similar to those of control astrocytes, contrasting with the more marked variations observed in A1. These findings suggest that lower-layer protoplasmic astrocytes, particularly those in the contusion core (A1), exhibit a more robust and distinct response to injury, likely due to significant reorganization and enrichment of cytoskeletal proteins. This morphological adaptation reflects the crucial role of protoplasmic astrocytes in modulating glial interactions and neural network function, as documented by their territorial organization into non-overlapping domains. Such domains are essential for regulating interactions with neural and non-neural cells and maintaining brain homeostasis [12, 42, 44, 58, 62]. Previous studies reported size-related variations in astrocyte domains that are regionally dependent [58, 62], which interact between them by the enrichment of membrane structures at their terminal branches expressing different channels, hemichannels and gap junctions to interchange molecules and signals [14, 34, 58]. In this work, we observed that protoplasmic reactive astrocytes at the contusion core (A1) were characterized by enriched primary and secondary branches and leaflets, as indicated by their Sholl intersections profile. This increased branching density could facilitate physical interactions within astrocyte domains influencing their ability to form barriers and segregate damaged from healthy tissue. These findings underscore the impact of astrocyte distribution among cortical layers and their proximity to the injury (contusion core) on their reactive responses.

Furthermore, our work pioneered a novel method for classifying astrocytes based on morphological changes in response to injury, correlating different morphological parameters with their intensity of the response. Previous works introduced this type of approaches to study neuronal subpopulations in the nucleus of the solitary tract [49] or to categorize diverse subpopulations of hippocampal astrocytes in migratory birds [2, 13, 28], reactive microglia in models of encephalitis [51, 54] or in models of neuroinflammation [20], and immune cells in starfishes [31]. Here, by employing a multimodality index (MMI), we selected relevant morphological parameters that best describe the population heterogeneity. Subsequently applying both hierarchical clustering (HC), and principal component analysis (PCA) we identified and constructed a dendrogram of reactive astrocyte responses. Thus, using a TBI analysis model we identified three main categories of astrocyte reactive responses: “moderate,” “strong,” and “very strong” (Fig. 5). This approach provided helpful cut-off values for each node level, offering insights into the diverse responses of reactive astrocytes. We observed an enrichment of sibling protoplasmic astrocytes in both “strong” and “very strong” reactive responses, as well as in fibrous, and pial astrocytes. This highlights the importance of lineage-specific responses in brain injury. Our previous studies demonstrated that sibling astrocytes may establish preferential coupling and form major-like domains [26], potentially sharing electrophysiological properties [25]. This analysis sheds some light on the complex relationship between astrocyte lineages, their clonal origin, and their varied reactions to traumatic brain injury.

Reactive astrocytes often exhibit morphological changes such as hypertrophy, increased expression of intermediate filaments or altered spatial distribution [18, 36, 52, 65]. Even these astrocytes, were classified as proliferative and non-proliferative, based on morphological changes [52]. These changes are typically associated with processes like neuroinflammation, synaptic remodeling, and neuroprotection in response to injury or disease. Our morphological reconstructions and categorization of reactive astrocytes provide quantitative insights into their spatial distribution and complexity revealing the intricate nature of their responses and potential impact on neural network dynamics following brain injury.

Further research integrating multiple techniques such as live-cell imaging, spatial omics, and functional assays are needed to create suitable datasets that comprehensively understand the relationship between astrocyte morphology and neural function. Recent research with machine learning demonstrated the value of such datasets for enhancing our understanding in basic science, computational modeling, and translational research [3]. By elucidating morphology-based reactive responses of astrocyte subpopulations, particularly those at the lesion core, we advance the understanding of astrocyte heterogeneity and its implications for neuroinflammation and tissue remodeling in brain injury contexts.

## Methods

### Animals

Animals were handled in compliance with the European Union guidelines on the use and welfare of experimental animals (2010/63/EU), and the Spanish legislation (Ministry of Agriculture, RD 1201/2005 and L32/2007). Procedures were approved by the CSIC Bioethical Committee and the Community of Madrid (Ref. PROEX 274.7.22) in Spain. We used isogenic C57Bl/6 J mouse pregnant females at the embryonic age 14 (E14) supplied by the stock from the Cajal Institute (Madrid, Spain). Animals were housed in standard cages, maintained under controlled light–dark cycles (12–12 hs) with food and water ad libitum. We made all efforts to minimize suffering and the number of animals used.

### StarTrack mixture and in utero electroporation

Cortical astrocytes were labeled using the StarTrack method as previously described [24]. StarTrack is a multicolor barcode genetic lineage tracing system, based on the PiggyBac system, that allows to tag single neural progenitors and follow their GFAP + progeny (glial cells). This system is composed of two elements, one, the DNA transposon vector that carries a gene encoding a fluorescent reporter protein (YFP, mKO, mCerulean, mCherry, Mt-Sapphire, and eGFP under the control of the human glial fibrillary acidic protein GFAP and the H2B promoter (only for nuclear labeling; and second, the transposase enzyme (CMV-hyPBase) which dimerizes the transposon at the ITR (Inverted Terminal Repeat) sequences, and guide and insert the fragment into areas of transcription (TTAA-AATT) in the genome (Fig. 1A). Thus, PiggyBac generates stable gene integration with high rates of transcription. GFAP StarTrack leads to consistent and higher levels of expression of the gene and produces the labeling of the cytoplasm and/or the nucleus of the cells.

Pregnant, C57BL/6j mice (P30-45) in gestational period E14 were deeply anesthetized with an isoflurane vaporizer system, Isova Vet, 2 ml/L, (4% induction, 2–3% maintenance, Centauro, Barcelona, Spain) and after conscious and pain assessment, were dissected the skin and abdominal tissues and exposed the uterine horns and embryos. Each embryo was carefully manipulated to receive an intraventricular injection of 2 µl of GFAP Startrack plasmid mixture (2–5 mg DNA/ml and 0,1% fast green solution to confirm the site of the injection and diffusion). Next, embryos were electroporated using an Electroporator ECM 830 system (BTX, Massachusetts, US) connected to 5 mm tweezer type electrodes (program: 1–2 trains, 5 square pulses, 35 V, 50 ms, followed by 950 ms intervals). Finally, uterine horns were placed back, and the abdominal tissues and skin were closed with absorbable suture 4/0 (Surgicryl, Hünningen, Berlin) and silk 4/0 (Lorca-Marin, Murcia, Spain). Postoperative care included antibiotic and analgesic administration (2.27% enrofloxacin, 5 mg/kg, Baytril, Bayer, Kiel, Germany and 0.5 mg/ml meloxicam, 300 μg/kg, Metacam, Boehringer Ingelheim Vetmedica GmbH, Rhein, Germany, subcutaneous) in controlled temperature environment (37 °C). Thus, neural progenitors of the subventricular zone were labeled with GFAP StarTrack plasmid mixture.

### Model of brain damage by traumatic brain injury

Next, the electroporated mice were subjected to a brain injury model in the somatosensory cortex where GFAP StarTrack astrocytes clones would develop a reactive response (detailed information in [38]). For this purpose, adult (P50, n = 2 or P90, n = 2) GFAP StarTrack mice were deeply anesthetized with an intraperitoneal injection of Equithesin (dose 0.33 ml/100 g, NIDA Pharmacy, Baltimore, MD, USA). After assessing conscious and pain response, they underwent to unilateral or bilateral penetrating lesion with a 22-gauge (0,7 mm) needle in the somatosensory cortex at the following stereotaxic coordinates (relative to Bregma) AP 0 and/or − 2 mm, ML 3.5 mm, DV–2 mm. Postoperative care included antibiotic and analgesic administration (2.27% enrofloxacin, 5 mg/kg, Bayer, Kiel, Germany and 0.3 mg/ml buprenorphine, 8 µg/kg, Buprex, Merck & Co., Inc., NJ, USA) in a controlled temperature environment (37 °C). Three to 5 days post-lesion (3–5dpl), mice were deeply anesthetized with intraperitoneal injection of sodium pentobarbital (40–50 mg/Kg, Dolethal, Vétoquinol Ltd, Buckingham, UK), transcardially perfused with 4% paraformaldehyde and post-fixed (overnight). Additionally, adult GFAP StarTrack (mCherry and YFP) mice (P30, n = 2) were used as controls under physiological conditions. Coronal sections of 50 µm were collected.

### Immunohistochemistry

Serial brain sections around the lesion core were rinsed several times with 1xPBS-0.1% Triton (0.1% PBST) and incubated in a blocking solution containing 5% normal goat serum NGS (5% NGS, 0.1% PBST) for 60 min, at room temperature. After the time, sections were incubated with selected primary antibodies, rabbit anti-Cux1 (1:300, Proteintech, 11733–1-AP); mouse anti-GFAP (1:500, Millipore/Thermo Fisher Scientific, MAB359); and mouse anti-Nestin (1:100, Cell Signalling, 4760); previously included in blocking solution (5% NGS, 0.1% PBST) overnight, at 2–8 °C. The next day, sections were rinsed (0.1% PBST) and incubated with the corresponding secondary antibody, goat anti-rabbit coupled to Alexa Fluor 488 (1:1000, Invitrogen/Thermo Fisher Scientific, A11008); and goat anti-mouse coupled to Alexa Fluor 568 (1:1000, Invitrogen/Thermo Fisher Scientific, A110004) in DAPI solution (1:10000, Sigma Aldrich Corporation, Saint Louis, USA) for 60–90 min, at room temperature. Finally, the sections were rinsed and mounted onto slides with aqueous solution (Fluoromount G, Electron Microscopy Sciences, EUA).

### Image acquisition

Image acquisition was performed at the confocal microscopy unit (Instituto Cajal-CSIC), using Leica TCS-SP5 (Houston, United States) and Leica STELLARIS 8 STED HM (Wetzlar, Germany) confocal microscopes. Imaging of GFAP astroglial populations was performed as previously described [23, 38]. Fluorescent proteins (XFP) were captured in separate channels. The wavelength of excitation (Ex) and emission (Em) for each XFP were: mT-Sapphire, Ex 405 nm, Em 520–535 nm, mCerulean, Ex 458 nm, Em 468–480 nm, EGFP, Ex 488 nm, Em 498–510 nm, YFP, Ex 514 nm, Em 525–535 nm, mKO, Ex 514 nm, Em 560–580 nm, and mCherry, Ex 561 nm, Em 601–620 nm (Leica TCS-SP5, 20x). Confocal laser lines were between 25 and 40% (Leica TCS-SP5, 20x) and the maximum projection images were created using LASAF Leica (v.3.02.16120, Leica Application Suite X, Leica Microsystems, Houston, United States) and ImageJ software (1.49v, http://rsbweb.nih.gov/ij).

### Clonal analysis

Clones of reactive astrocytes around the lesion were defined as those cells sharing fluorescent marks in the nucleus and the cytoplasm. We analyzed 281 cells corresponding to 35 clones of one GFAP StarTrack mouse. The analysis was conducted using ImageJ software (1.49v, http://rsbweb.nih.gov/ij). We assessed the percentage of clones per astroglial lineage, as well as the mean quantity, frequency, and dispersion of sibling cells within each astroglial lineage.

### Morphometric analysis

For the morphometric analysis, we analyzed 117 cells from five GFAP StarTrack electroporated mice subjected to brain injury (TBI group) and 45 cells from two GFAP StarTrack (mCherry and YFP) mice under physiological conditions (control group). We utilized the following measurement parameters: “area” (µm^2^), “convex hull area” (µm^2^), “perimeter” (µm), “circularity” (4π(“area”)⁄(“perimeter”)^2^, ratio), “solidity” (“area”⁄“convex hull area”, ratio). The simple neurite tracer (SNT) tool was employed to reconstruct and create a 3D mask of the cells, allowing us to quantify several parameters: “number of branches” (units), “length” (µm) “thickness” (relative to 0.05 threshold, µm^3^) “intersections” (units), “radius” (units) or 3D distance” (“4(radius)”, µm), and “complexity” (Sholl intersection profile, “intersections” as a function of “3D distance”, au). Additionally, we included Sholl components–“intersections”, “radius” and “complexity”- which correspond to the number of times a branch “intersects” imaginary concentric circles at a given “radius”, centered on the soma (Sholl intersections profile). As a result, Sholl analysis provides a one-dimensional profile (“complexity”) of astrocyte branching enabling comparison of branch density and spatial distribution [4, 8, 50].

### Reactive response categorization

Morphometric parameters were initially analyzed as separated features and subsequently grouped into categories of reactive astrocytes (adapted from [49]). First, multimodal index analysis (MMI) guided the selection of parameters for clustering. The MMI formula ([M3^2^ + 1]/[M4 + 3{(n-1)^2^/(n-2) (n-3)}]) include skewness (M3) and kurtosis (M4) to describe the shape of the data distribution curve. Next, we performed Hierarchical Clustering (HC) and Principal Component Analysis (PCA) on the cell population. Parameters were normalized using the Z-score method, where each value was adjusted by subtracting the mean of the parameter. For HC, we employed the Euclidean distance measure with a 2.3 distance threshold to calculate the length of the segment connecting two values. In PCA, principal components (PC) 1 and 2 were chosen, as they together explained approximately 80% of the morphological variance in the datasets. Finally, we constructed a logical tree of reactive astrocytes response-clusters that included cut-off values corresponding to a 95% confidence interval (CI).

### Statistical analysis

In the case of clonal and morphological analysis, we considered both absolute (such as the number of sibling cells per astroglial lineage, and comparisons of 2D and 3D parameters) and relative values (including parameter profiles, cell counts, and parameter contributions) for each selected parameter. For t-distributed Stochastic Neighbor Embedding SNE and clustering (HDBSCAN) analysis, we used a perplexity value = 5, and a minimum cluster size = 10 points. For the categorization of reactive astrocytes, we analyzed absolute values (clusters comparisons, and 2D and 3D parameters) and relative values (parameters profile, cells, and parameters contribution) for each selected parameter. Depending on the case, the graphs show mean, standard deviation (SD), standard error (SEM), and 95% confidence interval (CI). Statistical significance was assessed using one-tailed unpaired Student’s t test for comparison between two groups, and one-way or two-way ANOVA test for multiple comparisons, with post-hoc tests for pairwise comparisons (Bonferroni´s and Tukey´s test). Values with a confidence interval of 95% (p < 0.05) were considered statistically significant. Statistical analysis of the data and graphical representations were performed using GraphPad Prism (v 5.0, San Diego, United States) software, and R packages: Tidyverse (https://cran.r-project.org/web/packages/tidyverse/); Rtsne (https://cran.r-project.org/web//packages/Rtsne/Rtsne.pdf; https://github.com/jkrijthe/Rtsne); DBSCAN (https://cran.r-project.org/web/packages/dbscan/index.html); and Factoextra (https://cran.r-project.org/web/packages/factoextra/index.html).

### Supplementary Information

Supplementary Material 1.

Supplementary Material 2.

## Data Availability

Data generated during the current study are available from the corresponding author on reasonable request.
