# Supplementary figures and images for: Dissecting reactive astrocyte responses: lineage tracing and morphology-based clustering
Source: Biol Res. 2024 Aug 14;57:54. doi: 10.1186/s40659-024-00532-y (PMC11323641; doi:10.1186/s40659-024-00532-y)

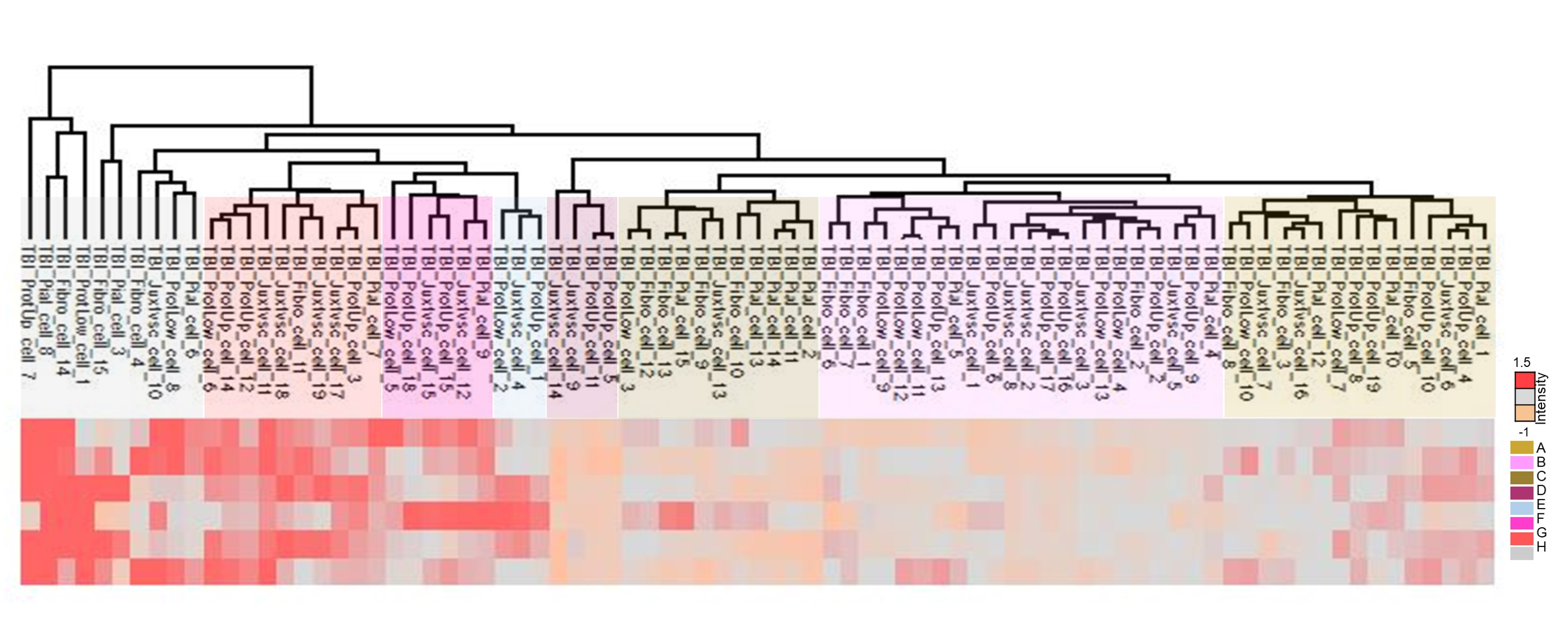

Supplement: Supplementary file 1 — Supplementary Material 1. [file 40659_2024_532_MOESM1_ESM.tif]

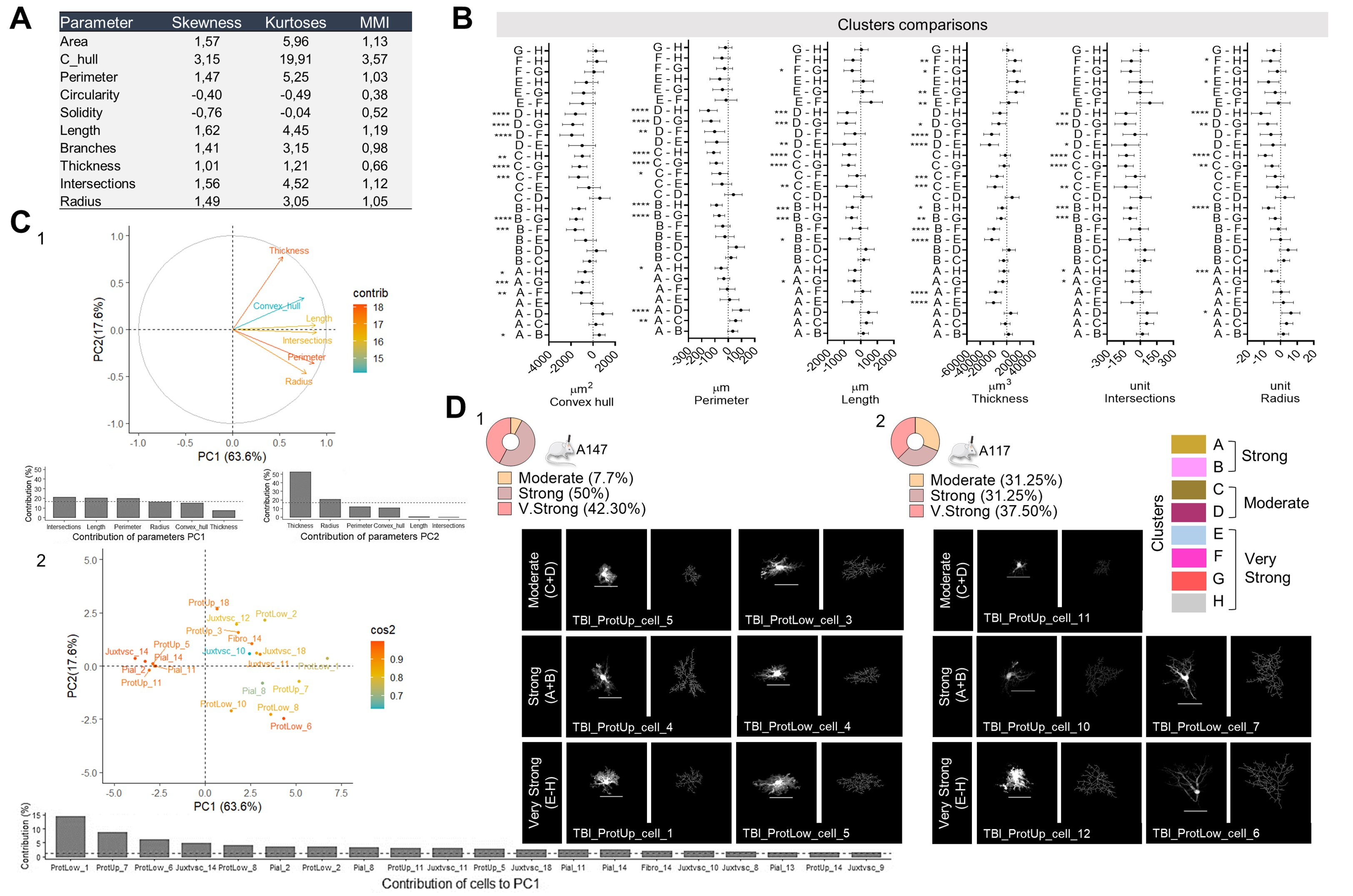

Supplement: Supplementary file 2 — Supplementary Material 2. [file 40659_2024_532_MOESM2_ESM.tif]
